# Identification of an antifungal lipopeptide from *Bacillus amyloliquefaciens* HAU3 inhibiting the growth of *Fusarium graminearum* using preparative chromatography and 2D-NMR

**DOI:** 10.1128/spectrum.00218-25

**Published:** 2025-08-28

**Authors:** Yuxuan Liu, Ziyao Shi, Xianhua Wan, Ruolin Wang, Yang Liu, Yujie Gong, Donghua Li, Yanhua Zhang, Hong Li, Guoxi Li, Xiaojun Liu, Xiangli Sun, Xiangtao Kang, Yanbin Wang

**Affiliations:** 1 College of Veterinary Medicine, Henan Agricultural University 70573, Zhengzhou, People’s Republic of China; 2 Key Laboratory of Livestock and Poultry Resources (Poultry) Evaluation and Utilization, Ministry of Agriculture and Rural Affairs 12654 , Zhengzhou, People’s Republic of China; 3 College of Animal Science and Technology, Henan Agricultural University 70573, Zhengzhou, People’s Republic of China; Connecticut Agricultural Experiment Station, New Haven, Connecticut, USA

**Keywords:** *Bacillus amyloliquefaciens*, *Fusarium graminearum*, silage, fengycin

## Abstract

The presence of fungal contamination and its mycotoxins in animal feed is pervasive, posing a significant threat to the well-being and performance of animals, as well as the safety of animal-derived food products. In this work, we screened a strain of *Bacillus amyloliquefaciens* (*B. amyloliquefaciens*) HAU3 that exhibits efficient antifungal activity against the growth of *Fusarium graminearum* (*F. graminearum*). The antifungal activity was detected in the supernatant, with 20% sterile supernatant demonstrating an impressive antifungal rate of 98.46% against *F. graminearum*. The antifungal activity of the strain was evaluated through spectrum analysis and silage trials, revealing its effective antifungal activity against multiple fungal species. Furthermore, the strain is capable of degrading ZEN and its derivatives. The targeted disruption of fungal mycelial membrane was observed using scanning electron microscopy and transmission electron microscopy. Additionally, staining with the reactive oxygen species (ROS)-sensitive fluorogenic dye DCFH-DA and propidium iodide (PI) revealed that the strain induces accumulation of ROS in fungal mycelia. The active compounds underwent further separation, purification, and detection. The prominent active peak was identified through mass spectrometry and magnetic resonance spectroscopy. The molecular structure of the active compounds was predicted to be lipopeptides composed of 8 amino acids known as fengycin. The whole genome sequencing and informatics analysis unveiled a total of 13 gene clusters responsible for the synthesis of secondary metabolites. The antifungal effects of *B. amyloliquefaciens* HAU3 are exerted through the synthesis of fengycin, which selectively targets and compromises the integrity of fungal mycelia membranes, thereby making it a potential biocontrol agent for mitigating mycotoxin contamination in feed.

**IMPORTANCE:**

Mycotoxin contamination in animal feed, predominantly driven by *Fusarium graminearum*, represents a persistent threat to livestock health and food chain integrity. Here, we report the isolation of a soil-derived *Bacillus amyloliquefaciens* HAU3, exhibiting potent and broad-spectrum antifungal activity alongside efficient biodegradation of zearalenone and its derivatives. Mechanistic dissection reveals that fengycin, the principal bioactive metabolite, compromises fungal membrane integrity and elicits intracellular oxidative stress, culminating in hyphal collapse. Genomic profiling uncovers a diverse repertoire of biosynthetic gene clusters underpinning secondary metabolite production. These findings establish strain HAU3 as a promising microbial chassis for the development of next-generation biocontrol strategies aimed at mitigating mycotoxin burden in agroecosystems.

## INTRODUCTION

Various pathogenic fungi, including the species of *Aspergillus*, *Fusarium, Penicillium*, *Alternaria*, and *Clostridium*, have the potential to infect crops during their growth in the field, leading to a reduction in crop yield and a deterioration in quality (1). Many of these fungi have the capacity to produce significant quantities of diverse mycotoxins under favorable conditions. The *Fusarium* species is renowned for synthesizing mycotoxins such as deoxynivalenol (DON), zearalenone (ZEN), and nivalenol (NIV), which are frequently detected in unprocessed agricultural commodities (2, 3). To date, over 500 toxic metabolites have been identified, with their chemical structures elucidated (4). The prevalence of fungal pathogen infections in field crops has been increasing due to global warming and the frequent occurrence of extreme weather events, leading to a rising trend in mycotoxin detection rates for both feed raw materials and finished feed (5, 6). The presence of these mycotoxins can significantly impair the quality and nutritional value of raw materials and animal feed, thereby exerting a detrimental impact on animal health and productivity by causing damage to vital organs such as the intestines, liver, and kidneys, resulting in various functional and metabolic disorders (7). Furthermore, the consumption of contaminated feed by livestock leads to the transfer of these toxins and their derivatives into animal-derived products, consequently posing a potential health risk to human consumers (4, 8). This interconnectedness emphasizes the urgent necessity for vigilance and effective implementation of management strategies to mitigate mycotoxin contamination throughout the entire food supply chain.

The investigation of functional microorganisms and enzymes for the elimination of mycotoxins has emerged as a prominent research area in addressing this issue (9). However, the simultaneous occurrence of diverse toxins and their derivatives in feed presents a challenge in addressing the comprehensive issue of mycotoxin contamination, as it cannot be resolved solely through the utilization of a single enzyme or microorganism due to catalytic specificity and specific reaction conditions (4, 10). Moreover, most toxins possess highly stable chemical structures that make it difficult to break them down in a timely manner prior to absorption under animal digestive tract conditions, thereby restricting the potential application of microorganisms and enzymes in this regard (11). Therefore, effectively inhibiting the growth and reproduction of fungi at their source remains crucial for mitigating the harm caused by mycotoxins.

The current practice involves implementing physical, chemical, and biological techniques in feed to effectively eradicate fungal contamination and impede mold growth and reproduction (12, 13). First, it is crucial to select premium-grade feed raw materials that are free from molds as a fundamental assurance for ensuring feed safety. By employing air elutriation (14), microwave irradiation (15), and polishing treatment (16) measures, the feed raw materials can be effectively processed to remove a majority of molds and their toxins on the surface of the grain. Dry treatment and low-temperature storage can significantly mitigate fungal proliferation in feed (17). The addition of preservatives such as organic acids or plant essential oils to feed is commonly employed for inhibiting fungal reproduction (18, 19); however, it may potentially impact the palatability and feed intake. Microorganisms in ecological environments often evolve specific strategies to suppress other microorganisms; hence, unveiling the underlying mechanism is expected to address the issue of mold and toxin pollution (20). The *Lactobacillus* genus utilizes SCFA production to lower the environmental pH levels along with bacteriocins that inhibit the growth and reproduction of other microorganisms, making it widely used in silage and fermented feed (21, 22). *Bacillus* species synthesize numerous secondary metabolites with antibiotic-like properties that antagonize mold and pathogen growth (23). Among these metabolites, fengycin, a lipopeptide first reported in July 1986 (24), has been recognized as a key antifungal agent due to its strong inhibitory activity against filamentous fungi. This unique property has positioned fengycin as an important component in biological control strategies for fungal contamination (25, 26).

In this study, *Fusarium graminearum* (*F. graminearum*) was employed as the indicator pathogen, and an aerobic strain with highly antifungal activity was isolated from soil samples collected from a wheat field. The isolated strain was identified as *Bacillus amyloliquefaciens* (*B. amyloliquefaciens*). Subsequent investigation revealed that the active compound responsible for its antifungal activity was present in the supernatant, whereas its antifungal mechanism involved specific targeting and disruption of the cell membrane of fungal hyphae. The active compound underwent separation and purification using HPLC, followed by identification through HPLC-MS and 2D-NMR techniques. Moreover, whole-genome sequencing was employed to predict biosynthetic gene clusters responsible for the production of secondary metabolites with potential antifungal activity.

## MATERIALS AND METHODS

### Materials

#### Sample sources

A total of 72 wheat soil samples were collected from eight locations in Henan Province (27). These samples were specifically collected during the wheat flowering stage to ensure temporal consistency. Soil was carefully extracted from within 5 cm around the wheat roots, with each sample weighing approximately 5 g. The collection process involved the use of a sterile spatula to minimize contamination, and the samples were immediately transferred into sterile sampling bags for subsequent analysis.

The fungal strains *F. graminearum*, *Simplicillium aogashimaense*, *Exserohilum turcicum*, *Sclerotium rolfsii*, *Bipolaris drechsleri*, and *Fusarium boothii,* which were previously preserved in our laboratory, were utilized in this study. These strains were maintained on PDA slants at 4°C and periodically subcultured to ensure viability.

#### Media and chemicals

The potato dextrose agar (PDA) medium, nutrient agar (NA), nutrient broth (NB), eosin-methylene blue (EMB) medium, and MRS liquid medium were procured from Huankai Microbial (Guangzhou, China). The CMC medium was purchased from Coolaber (Beijing, China). The potato dextrose water (PD) and Bacillus Physiological and Biochemical Identification kits were obtained from HaiBo Biologicals (Qingdao, China). Additionally, PBS (pH = 7.4), PI solution, and ROS assay kit were purchased from Solarbio (Beijing, China). Zearalenone (ZEN), α-zearalenol, and β-zearalenol (≥ 98% purity) were purchased from Pribolab (Qingdao, China).

### Methods

#### Screening and identification of bacterial strains

A 2 g sample of plant rhizosphere soil was collected and suspended in 20 mL of physiological saline solution. This mixture was then agitated at a speed of 160 rpm at 37°C for a duration of 2 h. The resulting bacterial solution underwent serial dilution ranging from 10^−1^ to 10^−7^, and subsequently, 100 µL from each dilution was spread onto nutrient agar plates. After 12 h of incubation at 37°C, a single bacterial colony was selected and subjected to repeated streaking for purification until microscopic examination confirmed the absence of contaminants. The purified strain was then preserved for subsequent experiments.

The isolated single bacterial colony was inoculated into nutrient broth and incubated for 12 h at 37℃ with agitation at 160 rpm to prepare a bacterial culture for subsequent antifungal assay. A mycelial plug of *F. graminearum*, measuring 5 mm in diameter, was centrally positioned on a PDA plate. Subsequently, 2 µL of the test bacterial culture was spot-inoculated onto PDA plates, located 2.5 cm away from the indicator fungus. Each isolated strain was tested in triplicate to ensure reproducibility. Following incubation in a constant temperature incubator at 25°C for 5 days, the antifungal effect was assessed, and the bacterial strain demonstrating the strongest antifungal activity was selected for further analysis.

The bacterial strain exhibiting the most potent antifungal effect was identified through comprehensive morphological, physiological, and biochemical tests, as well as 16S rRNA gene sequencing analysis. The bacterial cells in logarithmic phase were collected and fixed in 2.5% glutaraldehyde solution at 4°C for 4 h. After fixation, the samples were washed three times with PBS and subjected to gradient dehydration using ethanol solutions at concentrations of 10%, 25%, 45%, 60%, 80%, 90%, and 100%, with each step lasting 10 min. Subsequently, the samples were observed and photographed under SEM to examine the morphology of the bacterial cells. Add the bacterial suspension of strain HAU3 sequentially to the wells of the physiological and biochemical identification test strip, 100 µL per well, and place in a 37°C incubator to observe the reactions at regular intervals. The extraction of core genes was carried out using the state-of-the-art Bacterial Core Gene (UBCG) pipeline (28). Subsequently, these genes were concatenated, and a maximum-likelihood phylogenetic tree was constructed employing the Genetic Testing Registry (GTR) substitution model, as implemented in the RAxML tool (v. 7.0.4). The selection of 92 core genes was guided by an extensive data set consisting of 1,429 complete genome sequences representing 28 phyla, ensuring the inclusion of genes that are either broadly distributed across genomes or highly conserved as single-copy orthologs.

#### Evaluation of antifungal activity

##### Antifungal spectrum

Prior to experimental use, *Simplicillium aogashimaense*, *Exserohilum turcicum*, *Sclerotium rolfsii*, *Bipolaris drechsleri*, and *Fusarium boothii* were reactivated by transferring them onto fresh PDA plates and incubating them at 25℃ for 5 days. For the preparation for inoculation, each fungal strain was inoculated into CMC liquid medium under shaking conditions (150 rpm) at 28℃ for 3 days to generate spore suspensions. The resulting suspensions were filtered through sterile 400-mesh gauze, and the spore concentration was adjusted to 10^6^ spores/mL using a hemocytometer. For the antifungal activity evaluation, 100 µL of the spore suspension of the indicator strain was evenly spread onto PDA plates, ensuring a uniform distribution of the indicator fungus across the entire surface. Subsequently, 2 µL of the test strain culture was spot-inoculated at a fixed distance of 2.5 cm from the center of the plate. The plates were then incubated at 25℃ for 2 days, and the antifungal potential of the test strain against various plant pathogens was assessed by observing the formation of antifungal inhibition zones. The antifungal activity was calculated using the following formula: antifungal rate (%) = [(colony diameter of the control group – colony diameter of the treatment group)/(colony diameter of the control group – mycelial plug diameter)] × 100%.

##### Evaluation of antifungal activity in silage

The spores of *F. graminearum* were inoculated into CMC liquid medium and incubated at 25℃ with shaking at 150 rpm for 3 days to prepare a spore suspension. The resulting suspension was then filtered through sterile 400-mesh gauze to eliminate hyphal debris. The spore concentration was adjusted to 10^6^ spores/mL using a hemocytometer under a light microscope to ensure precise quantification prior to further application.

The experimental fields of Henan Agricultural University were utilized for wheat cultivation (longitude: 113.704663, latitude: 35.005322). The fields were divided into six plots, each approximately 100 m^2^ in size. Three plots were randomly selected and artificially inoculated with *F. graminearum* spores by spraying each wheat plant with 10 mL of a spore suspension (10^6^ spores/mL) using a hand-held sprayer to ensure uniform distribution. After infection treatment, wheat plants from the fungal-infected (FI) group and the non-infected (NFI) group were harvested separately and processed for subsequent experiments. The FI wheat samples were divided into two groups: the treatment group (FIHAU3), which was inoculated with the selected bacterial strain at a concentration of 1 × 10⁹ CFU per gram of silage material, and the control group (FIC), which was sprayed with an equal volume of physiological saline. The NFI group (NFIC) wheat samples were also sprayed with the same volume of physiological saline. The treated wheat silage was then stored in 5 L plastic silos, each sealed with two screw caps to maintain anaerobic conditions. The silos were kept at room temperature (25 ± 2℃). Sampling was conducted after ensiling periods of 7, 14, 30, and 60 days, with three replicates per group. Subsamples were taken from each silo under sterile conditions to ensure uniform microbial analysis.

To assess the microbial population, 20 g of the ensiled sample was homogenized with 180 mL of sterile physiological saline solution (0.85% NaCl) and subjected to shaking at 120 rpm for 2 h. The resulting suspension was then serially diluted (10^−1^, 10^−2^, 10^−3^, 10^−4^, 10^−5^, 10^−6^, and 10^−7^) and plated for microbial enumeration. Coliform bacteria were quantified using EMB agar, with incubation at 37°C for 1 day, whereas yeasts and molds were assessed using PDA, incubated at 28°C for 3 days.

### Degradation of ZEN and its derivatives by strains

*B. amyloliquefaciens* HAU3 was cultured in NB medium at 37°C with a shaking speed of 160 rpm for 12 h to prepare the fermentation broth. The bacterial culture was adjusted to a concentration of 1 × 10⁹ CFU/mL prior to further analysis. For the degradation assay, 900 µL of bacterial fermentation broth was mixed with 100 µL of ZEN, α-zearalenol, or β-zearalenol, resulting in a final concentration of 1 µg/mL. The mixtures were incubated at 37°C and 160 rpm for 24 and 48 h. After incubation, the cultures were centrifuged at 9,400 × *g* for 2 min, and the supernatant was filtered through a 0.22-µm membrane filter prior to HPLC analysis. HPLC analysis was conducted using an Agilent 1260 instrument equipped with an Amethyst C18-H column (4.6 × 250 mm, 5 µm). The mobile phase consisted of acetonitrile/water/methanol (46/46/8, vol/vol/vol), with a flow rate of 1 mL/min. The column temperature was maintained at 30°C, and detection was performed with excitation at 274 nm and emission at 440 nm. The injection volume was set at 10 µL, and each sample was analyzed in triplicate (*n* = 3). The degradation rate of ZEN and its derivatives was calculated based on peak area reduction compared with the untreated control.

### Localization and determination of antifungal active substances

#### The localization of antifungal active substances

A 1 mL aliquot of the microbial culture (1 × 10^9^ CFU/mL) was inoculated into PD medium and incubated at 37°C with shaking at 160 rpm for 24 h. After completion of incubation, the culture was centrifuged at 5,000 rpm for 10 min. The supernatant was then sterilized through filtration using a 0.22-µm filter to obtain a sterile supernatant (L2). The bacterial precipitate was resuspended in PBS. This resuspension underwent ultrasonic fragmentation at 400 W with cycles of 5 s on and off, lasting a total duration of 30 min. The sonication was performed on ice to prevent overheating. Following sonication, the sample was centrifuged at 4°C for 10 min at 5,000 rpm to separate the supernatant, which was collected to obtain the intracellular fluid (L1). The antifungal effects of L1 and L2 were assessed using the well diffusion method; 200 µL of each sample was added to wells with a diameter of 7 mm on PDA plates. The plates were incubated at 37℃ for 24 h, and the experiment was replicated three times under identical conditions.

#### Determination of antifungal efficacy

The antifungal efficacy of the bacterial strains was quantified using the agar dilution method. Specifically, a 1 mL aliquot of the microbial culture (1 × 10^9^ CFU/mL) was inoculated into PD medium and incubated at 37℃ with shaking at 160 rpm for 24 h. Subsequently, the bacterial sterile supernatant was obtained by centrifuging the cultures at 9,400 × *g* for 10 min, followed by filtration through a 0.22-µm membrane filter to ensure complete removal of cellular debris. Sterile supernatant was incorporated into PDA in varying proportions, with the supernatant comprising 5%, 10%, 15%, 20%, 25%, 30%, 40%, 50%, 60%, 70%, and 80% of the mixture, respectively. Control plates were prepared with PDA alone (without supernatant). A mycelial plug of *F. graminearum* with a diameter of 5 mm was then placed centrally on the PDA plates. These plates were incubated at a temperature of 25℃ for a duration of 5 days, with three replicates per treatment (*n* = 3). Fungal growth was measured daily by recording colony diameter (mm), and the percentage of antifungal activity was calculated relative to the control.

#### The observation of mycelial morphology using a scanning electron microscope and a transmission electron microscope

Equal quantities of *F. graminearum* mycelium (1 mg wet weight) were suspended in PBS solutions containing 0% (control) or 20% sterile supernatant, respectively. The suspensions were incubated at 25℃ for 48 h under gentle agitation. After incubation, mycelia were harvested by centrifugation, washed three times with PBS, and fixed in 2.5% glutaraldehyde at 4℃ for 12 h. Following fixation, mycelia were washed three times with PBS and subjected to a graded ethanol dehydration series consisting of 10%, 25%, 45%, 60%, 80%, 90%, and 100% ethanol, each for 10 min. Subsequently, the samples were post-fixed in 2% osmium tetroxide prepared in PBS (pH 7.4) within a dark chamber at 4℃ for 12 h, followed by further dehydration in ethanol. For SEM analysis, the dehydrated mycelia were sputter-coated with gold and observed using a scanning electron microscope SEM (Model Q45, Manufacturer Czech FEI). For TEM analysis, fixed mycelia were embedded in Epon resin, ultrathin sectioned (70–90 nm) using an ultramicrotome, stained with uranyl acetate and lead citrate, and examined under a TEM (Model HT7800, Manufacturer Hitachi).

#### PI staining and ROS detection

The hyphae of *F. graminearum* were harvested and adjusted to 1 mg wet weight per mL and then resuspended in PBS containing either 0% or 20% sterile supernatant. The suspensions were incubated at 25℃ for 48 h under gentle agitation. Following incubation, the hyphae were stained with 10 µg/mL PI in darkness at 25℃ for 15 min, followed by two washes with PBS to remove excess dye. Simultaneously, ROS detection was performed by incubating the hyphae with 10 µg/mL DCFH-DA in darkness at 37℃ for 20 min, followed by PBS washing to remove unbound probe. Finally, the treated hyphae were then examined under a fluorescence microscope to evaluate cellular integrity and the generation of ROS.

### Isolation, purification, and identification of antifungal active substances

The separation of lipopeptides was carried out using macroporous resin adsorption. A glass column (50 × 800 mm) was packed with approximately 60 cm of macroporous resin (Mitsubishi HP-20) prewashed with ethanol and subsequently rinsed with water until no ethanol odor remained. The fermentation broth was centrifuged at 9,400 × *g* for 10 min at 4℃, and the supernatant was filtered through a 0.45-µm membrane filter prior to being loaded onto the resin column. The column was initially washed with water, followed by elution with 10% ethanol to remove impurities. The target compounds were then eluted with 80% ethanol, and the eluates were collected. The collected eluates were concentrated via rotary evaporation at room temperature and subsequently lyophilized at −50℃ to obtain the crude extract.

The target compounds present in the crude extract were isolated utilizing a HANBON high-pressure preparative chromatography system, specifically the DAC100 model. The chromatography column employed was a DAC column with dimensions of 100 mm × 250 mm and a particle size of 10 µm. The chromatographic separation conditions were established as follows: the flow rate was maintained at 80 mL/min, with the mobile phase comprising component A: acetonitrile, and component B: an aqueous solution containing 0.1% acetic acid; an injection volume of 15 mL was utilized. A gradient elution method was implemented, wherein the proportion of mobile phase A increased from 15% to 100% over a duration of 70 min. Fractions corresponding to distinct peaks were collected into separate vials and subsequently concentrated via lyophilization to yield the target compound. The purified product underwent HPLC analysis to assess its purity and was further characterized using two-dimensional NMR spectroscopy on a Bruker AVANCE II instrument operating at 600 MHz, along with HPLC-MS for comprehensive molecular analysis.

### Whole genome sequencing and functional gene prediction

Bacterial genomic DNA was extracted using a commercial DNA extraction kit, and its quality was evaluated by assessing both purity and integrity. High-molecular-weight DNA was subsequently processed through end-repaired, barcoded labeling, and library preparation using the EXP-NBD104 kit. Fragmentation for the DNA (~3,500 bp) was achieved via ultrasonication, followed by end repair, A-tailing, and adapter ligation. The final sequencing library was quantified using Qubit (2 ng/µL), and the fragment size distribution was verified using an Agilent 2100 Bioanalyzer prior to sequencing. Whole-genome sequencing was performed on both Nanopore PromethION and Illumina NovaSeq PE150 platforms. Genome assembly was conducted using Unicycler (v0.4.8) with a hybrid approach that integrated long- and short-read sequencing data, achieving a total coverage depth of 100×. Biosynthetic gene clusters (BGCs) were predicted using antiSMASH (v4.0.2) (29), with default parameters for secondary metabolite analysis. The assembled bacterial genome sequence has been deposited in the NCBI GenBank database for public access.

### Statistical analysis

All statistical analyses were performed using SPSS (v26.0, IBM, USA). Data are presented as mean ± standard deviation (SD). A one-way ANOVA with Tukey’s *post hoc* test was used for multiple group comparisons. For the analysis of mold and coliform diversity during the silage process, a two-way ANOVA model was employed, incorporating two factors: days and inoculants, along with their interaction effects. When significant differences were observed, Tukey’s post hoc test was used for further comparisons. Independent samples *t*-tests were conducted for pairwise comparisons at each time point. Graphs were generated using GraphPad Prism (v8.4.0, GraphPad Software, USA), with significance levels denoted as *P* < 0.05 (*), *P* < 0.01 (**), and *P* < 0.001 (***).

## RESULTS

### Screening and identification of the strain

**Fig 1:**
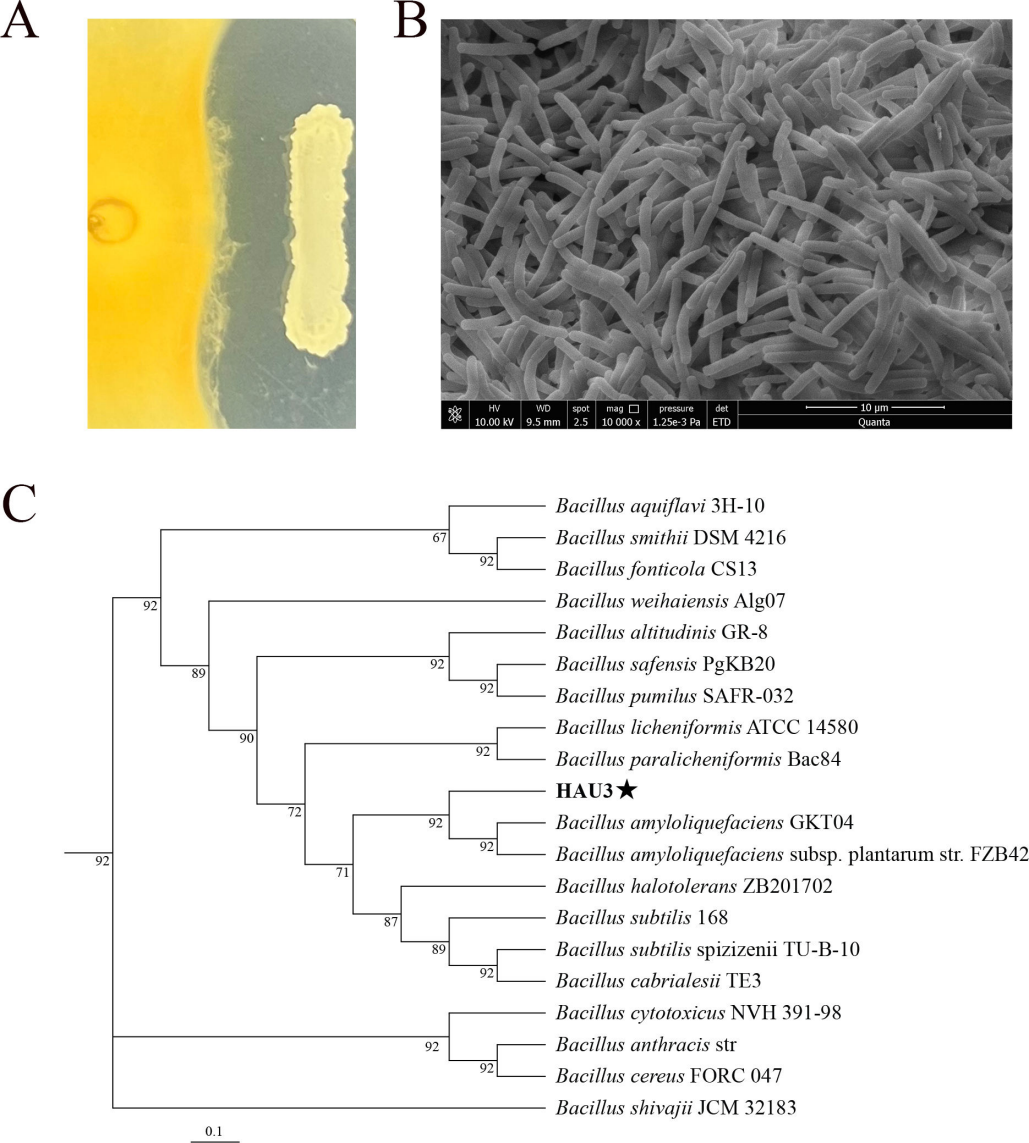
Screening, identification, and evaluation of strains. (A) The antagonistic activity of HAU3 against *F. graminearum*. (B) Scanning electron microscope of strain HAU3. (C) Phylogenetic tree of 16S rRNA PCR sequence of HAU3 strain.

To isolate strains exhibiting antifungal activity against *F. graminearum*, an *in vitro* dual-culture assay was conducted. A total of 33 strains were screened from wheat rhizosphere soil samples, all of which demonstrated significant antifungal activity against *F. graminearum* mycelial growth (Fig. S1 and Table S1). Among these strains, the one with the most potent antifungal effect was selected for further investigation and designated HAU3, with an antifungal inhibition zone diameter measuring up to 5.44 mm (Fig. 1A). Scanning electron microscopy analysis revealed that HAU3 is a short rod-shaped bacterium approximately 6 µm in length (Fig. 1B). Based on the maximum-likelihood phylogenetic tree constructed from 92 core genes, strain HAU3 was clustered in the same clade as *B. amyloliquefaciens* (Fig. 1C). In conjunction with physiological and biochemical characterization (Table S2), such as glucose (+), starch hydrolysis (+), Gram staining (+), and V-P test (+), the strain was identified as *B. amyloliquefaciens*.

### Evaluation of antifungal activity against multiple fungal species

#### Antifungal spectrum

**Fig 2:**
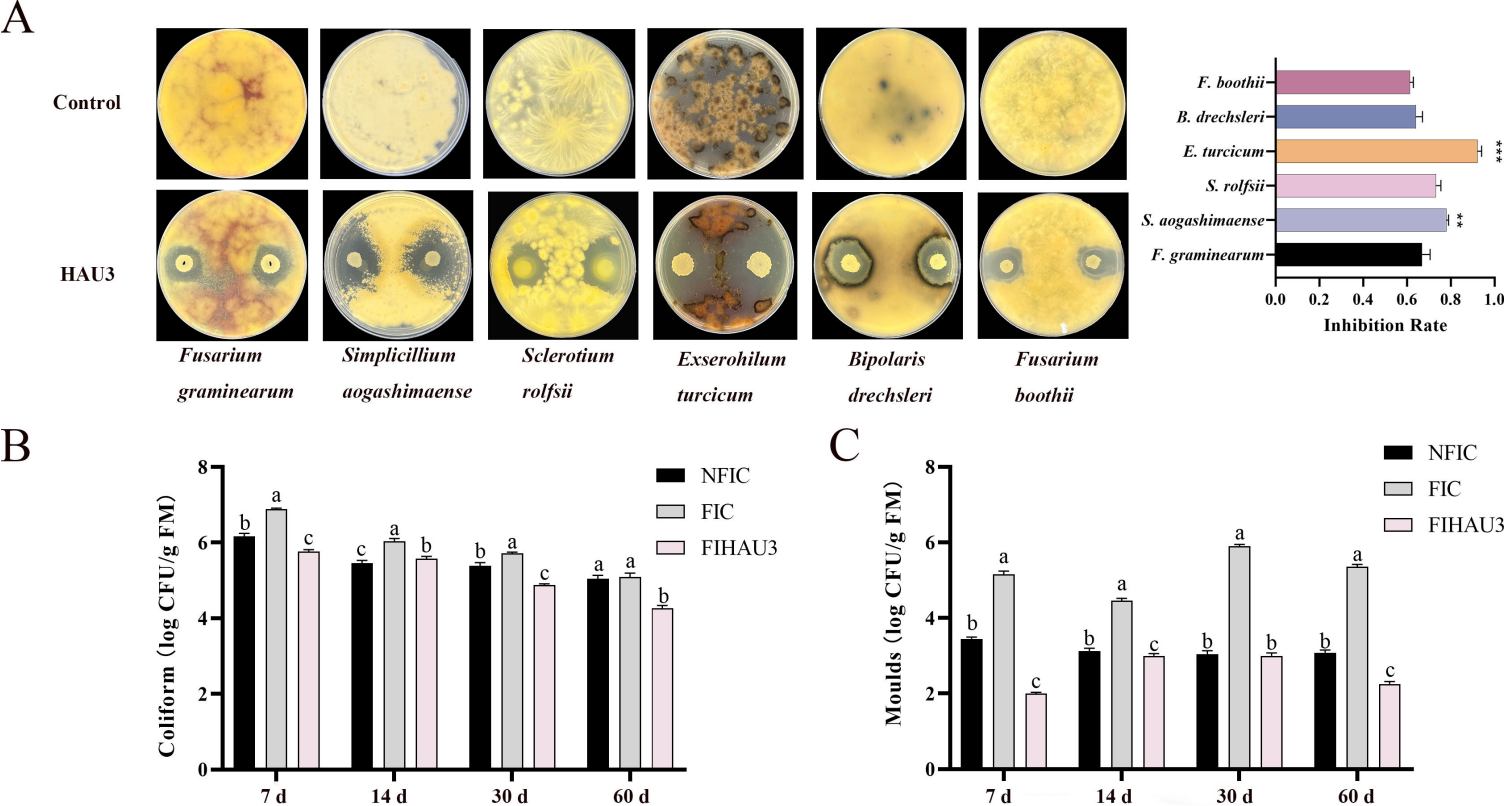
Evaluation of antifungal activity against multiple fungal species. (A) Antagonistic activity of HAU3 against different plant pathogenic fungi. (B) Molds counts in silages during the fermentation process.

In addition, the analysis of the antifungal activity against various plant pathogenic fungi showed that strain HAU3 exhibited antifungal rates of 66.94%, 78.06%, 73.32%, 92.25%, 64.12%, and 61.47% against *F. graminearum*, *S. aogashimaense*, *S. rolfsii*, *E. turcicum*, B. drechsleri, and *F. boothii*, respectively. Notably, strain HAU3 demonstrated significantly higher antifungal rates against *S. aogashimaense* and *E. turcicum* compared with *F. graminearum* (Fig. 2A).

#### Evaluation of antifungal activity in silage

The results indicate that throughout the ensiling process, the counts of coliform and molds in the *B. amyloliquefaciens* HAU3 group consistently remained lower than those observed in the FIC groups (*P* < 0.05). (Fig. 2B and C). At days 7, 30, and 60, the coliform count in the FIHAU3 group was significantly lower than that in the NFIC group (*P* < 0.05), whereas at day 14, it was significantly higher than in the NFIC group (*P* < 0.05) (Fig. 2B). At days 7, 14, and 60, the mold count in the FIHAU3 group was significantly lower than that in the NFIC group (*P* < 0.05). At day 30, there was no significant difference in mold count between the FIHAU3 and NFIC groups (*P* > 0.05) (Fig. 2C).

### Degradation of ZEN and its derivatives by strains

**Fig 3:**
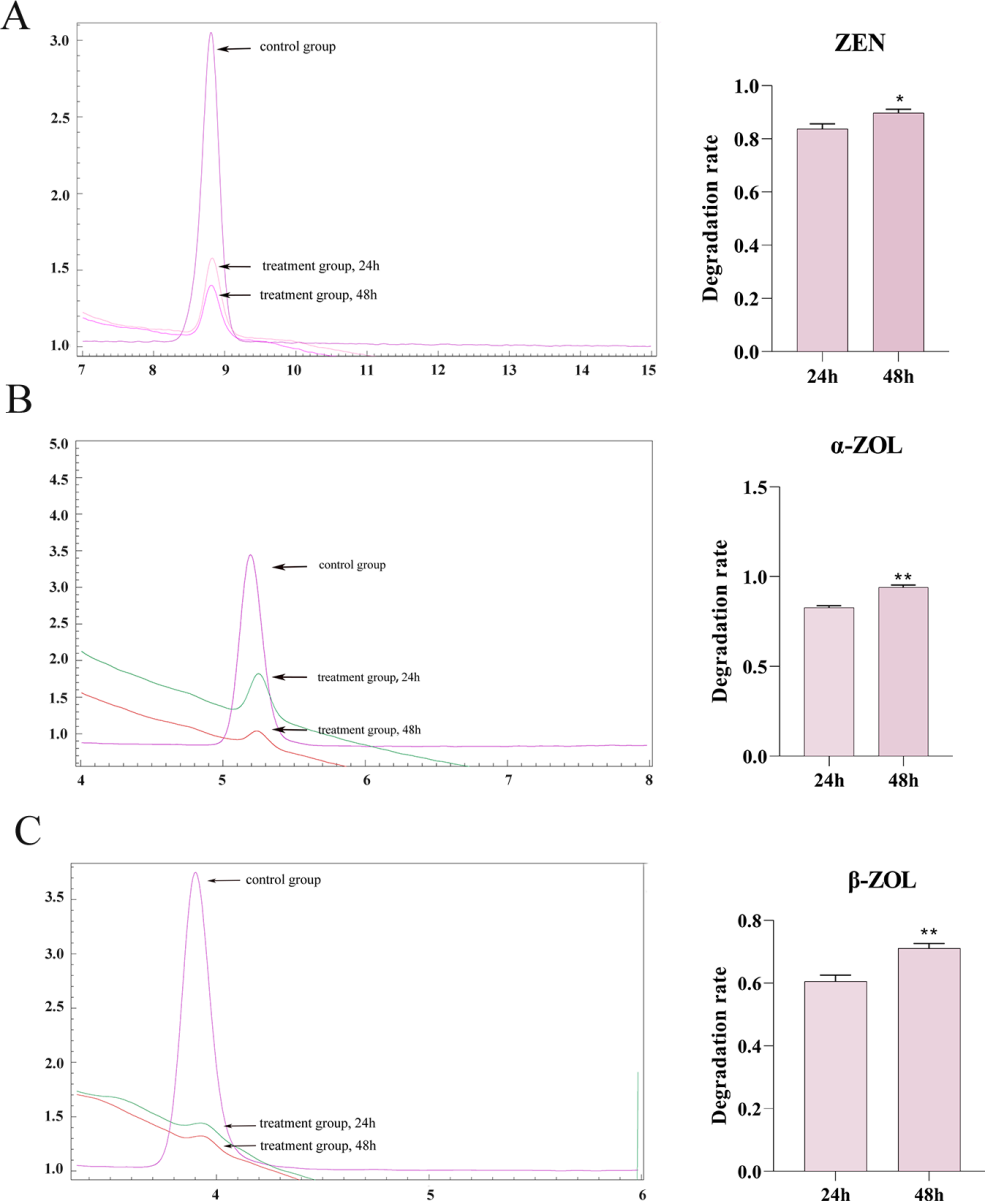
Degradation of ZEN and its derivatives by B. amyloliquefaciens HAU3. (A) ZEN. (B) α-ZOL. (C) β-ZOL.

HPLC results are shown in Fig. 3. The degradation rates of the HAU3 strain for zearalenol, α-zearalenol, and β-zearalenol were 83.75%, 82.80%, and 60.57% at 24 h, and 89.73%, 94.02%, and 71.13% at 48 h, respectively.

### Localization and determination of antifungal active substances

#### The localization of antifungal active substances

**Fig 4:**
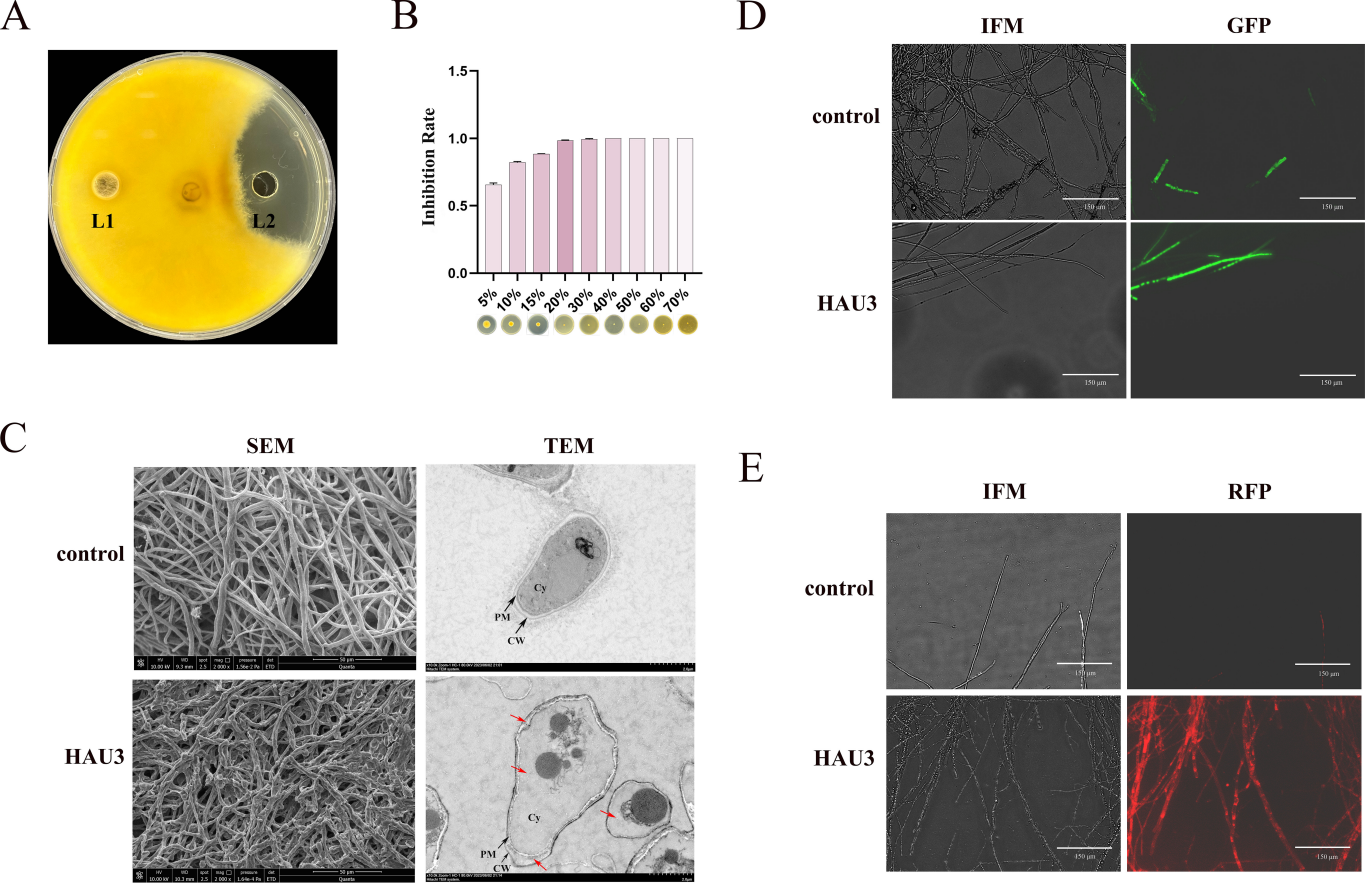
Evaluation of antifungal effects and target analysis. (A) Localization of the antifungal substance in HAU3; L1 was the supernatant of the intracellular extract; and L2 was the supernatant of sterile. (B) The inhibitory effect of supernatants with different concentrations on *F. graminearum*. (C) Transmission electron micrographs and scanning electron microscopes of plasma membrane changes caused by HAU3 treatments in *F. graminearum*. (D) Detection of ROS was based on DCFH-DA staining after treatment with HAU3. (E) Detection of *F. graminearum* viability based on propidium iodide staining after treatment with HAU3.

The intracellular fluid (L1) and the sterile supernatant (L2) obtained from *B. amyloliquefaciens* HAU3 were collected and assessed for their antifungal effects on the growth of *F. graminearum*. The outcomes demonstrated that L1 did not exhibit antifungal activity against the mycelial proliferation of *F. graminearum*, as illustrated in Fig. 4A.

#### Evaluation of the antifungal effect of sterile supernatants

Evaluating the antifungal impact of strain HAU3 on *F. graminearum* serves as a means to investigate the influence of HAU3 on the growth of toxin-secreting fungi. The introduction of a 20% concentration of sterile supernatant derived from strain HAU3 engendered an antifungal rate of 98.46% (Fig. 4B). Consequently, all subsequent experimental procedures employed this 20% concentration of sterile supernatant as a basis for further antifungal investigations.

#### Scanning electron microscope and transmission electron microscope observations of mycelial morphology

Scanning electron microscopy revealed that the mycelium and spores of the control group manifested a regular and intact surface configuration. In contrast, the treatment group presented wrinkled mycelium accompanied by evidence of intracellular material efflux, suggesting cell wall disruption. Transmission electron microscopy further expounded the ultrastructural modifications in *F. graminearum* mycelium subsequent to treatment with *B. amyloliquefaciens* HAU3. The mycelium from the control group demonstrated a uniform and readily distinguishable cell wall thickness along with homogeneous cytoplasmic density. Conversely, the treated group exhibited compromised cell wall integrity, characterized by the leakage of intracellular contents and an irregular cell wall lacking distinct strata and manifesting non-uniform thickness (Fig. 4C).

#### ROS detection and PI staining

DCFH-DA facilely permeates the cytoplasmic membrane and is hydrolyzed by intracellular esterases to generate DCFH upon intracellular penetration. Subsequently, intracellular ROS engage in interaction with DCFH, giving rise to the emission of green fluorescence. Figure 4D illustrates that the mycelium treated with *B. amyloliquefaciens* HAU3 manifested a markedly augmented green fluorescence compared with the control group, indicating elevated ROS levels.

Propidium iodide (PI) is non-permeable to viable cells with intact membranes; however, it can permeate and intercalate with DNA in cells manifesting impaired membrane integrity, thereby giving rise to red fluorescence. The figure demonstrates that untreated *F. graminearum* mycelium evinced negligible red fluorescence. In contrast, subsequent to treatment, there was a substantial increase in the proportion of mycelial emitting red fluorescence (Fig. 4E).

### Isolation, purification, and identification of antifungal active substances

**Fig 5:**
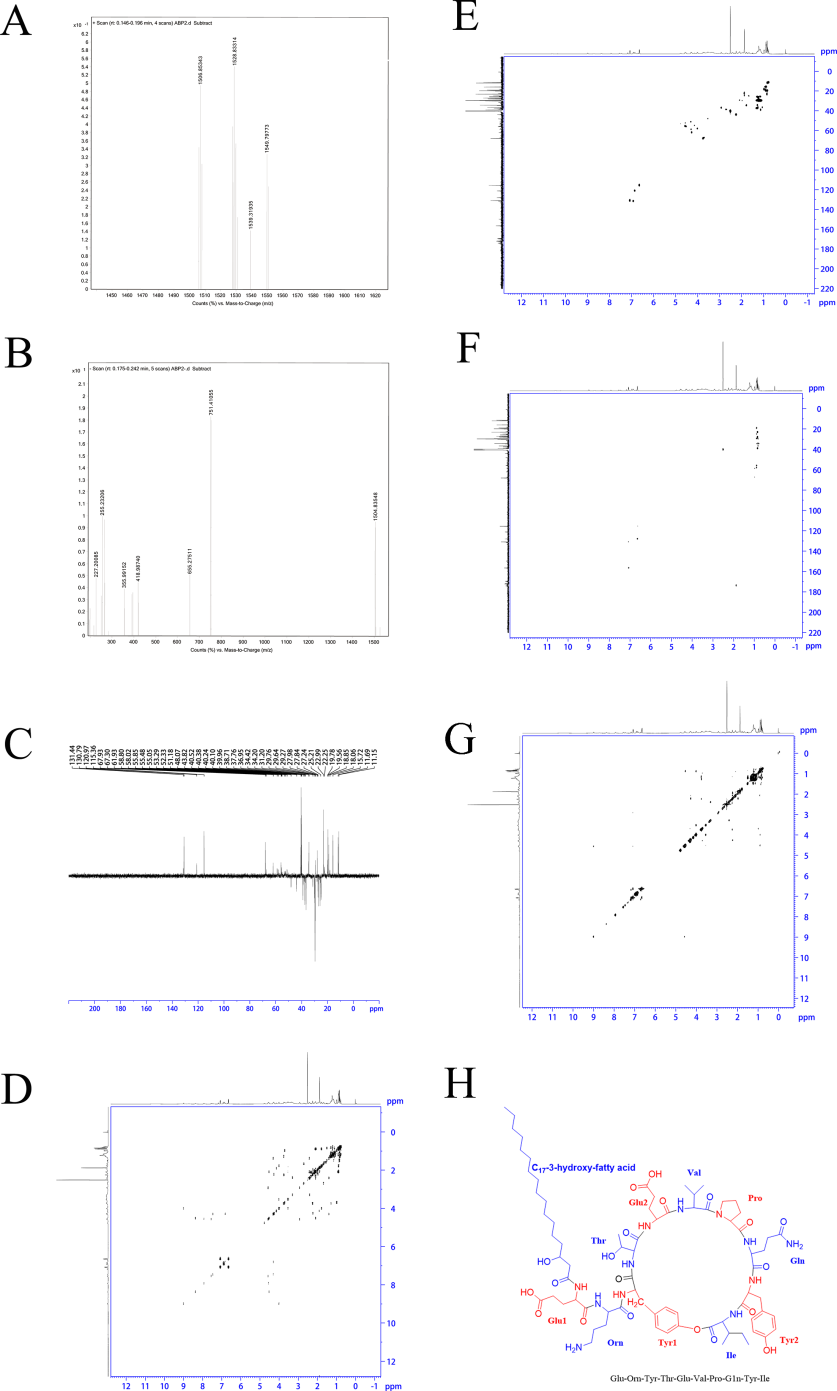
Structure identification of antimicrobial compounds. (A) Determination of antimicrobial peptide extracted from HAU3 using HPLC-MS (positive ion scanning mode). (B) Determination of antimicrobial peptide extracted from HAU3 using HPLC-MS (negative ion scanning mode). (C) DEPT 135 NMR spectrum; (D) COSY NMR spectrum. (E) HSQC NMR spectrum. (F) HMBC NMR spectrum. (G) ROESY NMR spectrum. (H) The structure of fengycin.

To further elucidate the antifungal mechanism of *B. amyloliquefaciens* HAU3, we performed a preliminary extraction of its antifungal compounds and subsequently purified multiple active components with confirmed antifungal activity (Fig. S2A). Among these, Component 2 demonstrated the most potent antifungal effect and was therefore selected for in-depth analysis. HPLC results indicated that Component 2 achieved a purity level of 99% (Fig. S2B). HPLC-MS analysis of component 2 disclosed a mass-to-charge ratio (m/z) of 1506.85343 in the positive ion scan (Fig. 5A) and 1504.83548 in the negative ion scan (Fig. 5B). On the basis of these data, we postulated that the molecular weight of the compound is approximately 1505.8. The precise molecular weight indicated by an m/z value of 751.41055 in the negative ion scan implies that it corresponds to a [M-2H]^2-^ ion with an exact molecular weight of 1504.8 Da. Additionally, 2D NMR spectroscopy, encompassing COSY, HSQC, HMBC, and DEPT135, suggested that ABP2 is a glycosylated lipopeptide composed of an eight-amino acid chain (Fig. 5C through F). ROESY correlation analysis further substantiated that the configuration of ABP2 is congruent with that of the inhibitory lipopeptide known as fengycin (Fig. 5G).

### Whole genome sequencing analysis

The comprehensive genome of strain HAU3 encompasses 4,063,259 bp, featuring a GC content of 46.27%. It constitutes 4,246 protein-coding DNA sequences, 86 tRNA genes, and 27 rRNA operons (Fig S3A). AntiSMASH analysis discerned 13 gene clusters within the *B. amyloliquefaciens* HAU3 genome that are associated with secondary metabolite biosynthesis (Table S3). These encompass one non-ribosomal peptide synthetase (NRPS), one polyketide synthase-like (PKS-like), two terpene synthases, one class-II lanthipeptide synthetase, two trans-acyltransferase polyketide synthases (transAT-PKS), one hybrid transAT-PKS, one type III PKS (T3PKS), one hybrid NRPS, one betalactone cluster, and one functionally uncharacterized cluster. Notably, cluster 8 exhibits complete amino acid sequence homology with known clusters accountable for the synthesis of fengycin (Fig. S3B).

## DISCUSSION

The frequent occurrence of fungal contamination in field crops (30), attributed to the optimal temperatures and humidity conditions (31, 32), poses a significant threat to downstream farmed animals along the food chain due to mycotoxin presence in feed raw materials. However, limitations associated with pesticide use for preventing and controlling fungal hazards in agricultural fields include the development of drug resistance and potential accumulation of pesticide residues (33, 34). Therefore, employing the microbial antifungal mechanism within the ecological environment to suppress fungal growth represents an eco-friendly, sustainable, and secure approach (35–38). In this regard, the Bacillus genus stands out for its robust stress resistance and abundant metabolite production, exhibiting potent antifungal activity against pathogens and establishing it as an efficacious biocontrol agent (39, 40). In this study, a strain of *B. amyloliquefaciens* HAU3 was isolated and screened from soil samples collected from wheat fields. This strain exhibited remarkable antifungal effects on the growth of *F. graminearum*, with an antifungal rate of 98.46% observed in the 20% sterile supernatant.

To assess the antifungal efficacy of *B. amyloliquefaciens* HAU3 under real agricultural conditions, we simulated a high-humidity and high-temperature environment conducive to fungal infection. Previous studies have shown that silage additives can effectively inhibit pathogenic bacteria by lowering pH levels (41), whereas inoculants can reduce mold and coliform counts during ensiling (42, 43). Our findings demonstrate that treatment with *B. amyloliquefaciens* HAU3 significantly diminishes the presence of molds and coliforms in silage, likely attributed to its capacity for producing multiple antifungal peptides. However, as ensiling progresses into later stable stages characterized by decreased pH levels and establishment of an anaerobic environment, the strain itself may also be inhibited.

The ultrastructure of *F. graminearum* was investigated via scanning electron microscopy and transmission electron microscopy to further explore the underlying antifungal mechanism and target site. Our findings demonstrate that *B. amyloliquefaciens* HAU3 exerts antifungal activity by disrupting the fungal cell membrane, leading to mycelium shrinkage, cytoplasm leakage, and irregular cell wall structure. These observations are in line with previous reports of Huang et al. (44). In the broader context of cellular biology, reactive oxygen species (ROS) play a dual role: at lower concentrations, they act as vital signaling molecules that regulate numerous molecular processes (45); at higher levels, they can inflict extensive cellular damage via DNA disruption, lipid peroxidation, and protein oxidation, ultimately resulting in cell death (46). Xie et al. demonstrated that *B. amyloliquefaciens* OR30 inhibits the growth of *F. graminearum* by inducing ROS accumulation and subsequent hyphal cell death through the production of lipopeptide antibiotics (47). Similarly, Farzand et al. reported that *B. amyloliquefaciens* FZB42 effectively suppresses *Sclerotinia sclerotiorum* by inducing systemic resistance (ISR) and modulating antioxidant pathways via the production of fengycin (48). Intriguingly, our study disclosed that *B. amyloliquefaciens* HAU3 induced a considerable accumulation of ROS within the mycelium of *F. graminearum*. Subsequent propidium iodide staining assays validated the ability of *B. amyloliquefaciens* HAU3 to induce cell death in the mycelium of *F. graminearum*. This combination of ultrastructural analyses with molecular insights offers a detailed understanding of the fungicidal action of *B. amyloliquefaciens* HAU3, highlighting its potential as a biocontrol agent in addressing fungal contamination in agricultural scenarios.

The genomes of Bacillus species are abundantly endowed with biosynthetic gene clusters (BGCs) encoding bioactive secondary metabolites, which primarily contribute to their bioactivity through the production of a diverse array of chemical compounds (49). In recent decades, numerous secondary metabolites exhibiting antifungal properties have been identified (50, 51). However, the regulatory mechanisms governing the synthesis of these antifungal compounds remain largely elusive. In this study, we mined the complete genome information of *B. amyloliquefaciens* HAU3 to identify potential secondary metabolite BGCs that may be involved in exerting antifungal activity. Several BGCs exhibited significant similarity to known bioactive compounds, suggesting functional conservation within these clusters. For example, cluster6, cluster7, and cluster11 displayed 100% similarity to the BGCs responsible for macrolactin H, bacillaene, and difficidin synthesis, respectively, indicating their potential involvement in the synthesis of these broad-spectrum antimicrobial compounds. These metabolites have demonstrated substantial inhibitory effects against gram-positive pathogens, particularly in terms of biofilm inhibition and biocontrol applications (52). Furthermore, cluster 8 and cluster 12 were found to be 100% identical to the gene clusters associated with fengycin and bacillibactin production. Studies have shown that fengycin acts as a cyclic lipopeptide antibiotic, effectively inhibiting plant pathogenic fungi, whereas bacillibactin functions as a siderophore, facilitating microbial competition under iron-limited conditions, thereby potentially providing a survival advantage to the host within microbial communities (53, 54). In contrast to these highly similar BGCs, certain clusters (e.g., cluster 1 with only 35% similarity to locillomycin and cluster 3 with 7% similarity to butirosin A/B) exhibit low similarity to known compounds. This disparity in similarity suggests that these BGCs may synthesize structurally related but functionally distinct secondary metabolites. Previous studies indicate that low-similarity BGCs often lead to the discovery of novel bioactive molecules with potential applications in antibiotic, anticancer, and antifungal drug development. However, further chemical characterization and functional assays are needed to validate the biological activity of these gene clusters. Moreover, several BGCs showed no significant similarity to any known compounds in the MIBiG database, including cluster 4, cluster 5, cluster 9, and cluster 10, indicating that these clusters may represent novel biosynthetic pathways and unique compound types. Notably, cluster 5, a lantipeptide class II gene cluster, has garnered considerable attention in antimicrobial peptide research as a promising candidate against drug-resistant pathogens (55). Further investigation into these unmatched BGCs could reveal their significance in microbial physiology and ecological adaptation while potentially advancing the development of new antimicrobial agents.

To further validate the functionality of secondary metabolite gene clusters identified through genome mining, we employed preparative chromatography to isolate and purify potential antifungal compounds from *B. amyloliquefaciens* HAU3. Multiple fractions exhibited inhibitory effects against *F. graminearum*, with the most potent fraction being identified as fengycin. Fengycin, a fungicide, has demonstrated significant antifungal activity (56). It interacts with the lipid bilayer of cell membranes, modifying their structure and permeability, thereby conferring resistance against various pathogens while safeguarding the plant host and the producing bacteria, highlighting its promising potential for application (57). Interestingly, other isolated fractions also displayed antifungal effects, though their efficacy was notably lower than that of fengycin. We speculate that these components may share similar molecular structures while exhibiting variations in substrate characteristics, such as carbon chain length and amino acid composition, thereby resulting in structurally analogous variants. Belbahri et al. proposed that the genomic plasticity in metabolic pathways of different strains of *B. amyloliquefaciens*, as they adapt to diverse environments, results in a diversity of secondary metabolite production (58). This diversity may stem from variations in substrate and the influence of the regulatory gene, leading to structurally similar yet compositionally distinct metabolites. In complex microbial ecosystems, where nutrient availability and the constraints of catalytic enzymes are significant factors, structurally similar secondary metabolites with distinct molecular compositions may be produced under the regulation of the same gene cluster. Sun et al. demonstrated that by knocking out the regulatory gene rapC in strain fmbJ, gene regulation within metabolic pathways can significantly enhance the production yield of lipopeptide bacillomycin D, suggesting that enzyme specificity within a shared gene cluster can influence both the structural characteristics and quantity of the metabolites (59). Additionally, Scholz et al. discovered that *B. amyloliquefaciens* FZB42 synthesizes a novel cyclic bacteriocin (amylocyclicin) in different ecological niches, resulting in various molecular variants due to its cyclic structure being influenced by specific conditions (60).

### Conclusion

The strain of *B. amyloliquefaciens* HAU3, which possesses the capability to effectively inhibit the growth of *F. graminearum*, was isolated and evaluated. The result demonstrated that a 20% sterile supernatant derived from this strain exhibited a remarkable antifungal efficacy of 98.46% in suppressing *F. graminearum*. The investigation ascertained that the strain exerts its antifungal activity by compromising the integrity of the cell membrane and inducing the accumulation of ROS. The subsequent isolation and identification of the antifungal substance unveiled fengycin as the compound accountable for this inhibitory activity.

## Data Availability

The raw sequence data obtained in this study were deposited in the NCBI Sequence Read Archive (SRA) under the accession number PRJNA1077085.

## SUPPLEMENTAL MATERIAL

Supplemental material spectrum.00218-25-s0001.pdf Tables S1 to S3; Fig. S1 to S3.

ASM does not own the copyrights to Supplemental Material that may be linked to, or accessed through, an article. The authors have granted ASM a non-exclusive, world-wide license to publish the Supplemental Material files. Please contact the corresponding author directly for reuse.
