# Supplementary material for: Identification of an antifungal lipopeptide from Bacillus amyloliquefaciens HAU3 inhibiting the growth of Fusarium graminearum using preparative chromatography and 2D-NMR
Source: Microbiol Spectr. 2025 Aug 28;13(10):e00218-25. doi: 10.1128/spectrum.00218-25 (PMC12502639; doi:10.1128/spectrum.00218-25)
Supplement: Supplemental material — Tables S1 to S3; Fig. S1 to S3. [file spectrum.00218-25-s0001.pdf]

Supplementary Table 1：Inhibition Zone Diameters of Selected Strains Against *F. graminearum*.

Strain number	Inhibition zone diameter（mm）	Strain number	Inhibition zone diameter（mm）	Strain number	Inhibition zone diameter（mm）
HAU1	3.13±0.04	HAU12	5.11±0.02	HAU23	4.52±0.10
HAU2	4.72±0.05	HAU13	3.51±0.03	HAU24	3.52±0.05
HAU3	5.44±0.05	HAU14	2.47±0.05	HAU25	4.69±0.03
HAU4	4.28±0.07	HAU15	4.40±0.04	HAU27	5.17±0.06
HAU5	3.08±0.11	HAU16	4.42±0.04	HAU29	2.08±0.12
HAU6	4.09±0.08	HAU17	3.86±0.10	HAU30	3.90±0.02
HAU7	3.05±0.08	HAU18	3.83±0.05	HAU31	2.99±0.09
HAU8	3.96±0.08	HAU19	3.78±0.04	HAU32	2.76±0.06
HAU9	4.62±0.10	HAU20	3.45±0.13	HAU34	5.34±0.06
HAU10	4.63±0.06	HAU21	3.57±0.23	HAU35	4.96±0.05
HAU11	2.62±0.10	HAU22	4.52±0.04	HAU36	5.30±0.03

Supplementary Table 2: Physiological and biochemical identification of *B. amyloliquefaciens* HAU3

Item	Result	Item	Result
Gram Staining	+	Starch Hydrolysis	+
Cell Morphology	Short Rod-Shaped	D-Xylose	+
Spore Formation	+	L-Arabinose	+
PH5.7	+	VP Test	+
Nitrate Reduction	-	Citrate Utilization	+
Propionate	-	D-Mannitol	-
7% NaCl	+	Gelatin Liquefaction	+

Supplementary Table 3: Secondary Metabolite Biosynthetic Gene Clusters of *Bacillus amyloliquefaciens* HAU3.

**Cluster**	**Type**	**From**	**To**	**Most similar known cluster**	**Similarity**	**MIBiG BGC**
cluster1	NRPS,transAT-PKS	195,511	273,065	locillomycin/locillomycin B/locillomycin C	35%	BGC0000426
cluster2	NRPS	343,161	407,971	surfactin	82%	BGC0000433
cluster3	PKS-like	977,370	1,018,614	butirosin A/butirosin B	7%	BGC0001928
cluster4	Terpene	1,103,492	1,120,827	-	-	BGC0000846
cluster5	lanthipeptide-class-ii	1,240,080	1,268,969	-	-	BGC0000528
cluster6	transAT-PKS	1,432,968	1,520,793	macrolactin H	100%	BGC0000181
cluster7	transAT-PKS，T3PKS,NRPS	1,740,298	1,840,900	bacillaene	100%	BGC0001089
cluster8	NRPS,transAT-PKS,betalactone	1,906,145	2,042,215	fengcyin	100%	BGC0001098
cluster9	terpene	2,065,799	2,087,682	-	-	BGC0002173
cluster10	T3PKS	2,156,326	2,197,426	-	-	BGC0000282
cluster11	transAT-PKS	2,459,865	2,553,641	difficidin	100%	BGC0000176
cluster12	NRP-metallophore,NRPS,RiPP-like	3,164,265	3,216,058	bacillibactin	100%	BGC0000616
cluster13	other	3,728,858	3,770,276	bacilysin	100%	BGC0000888

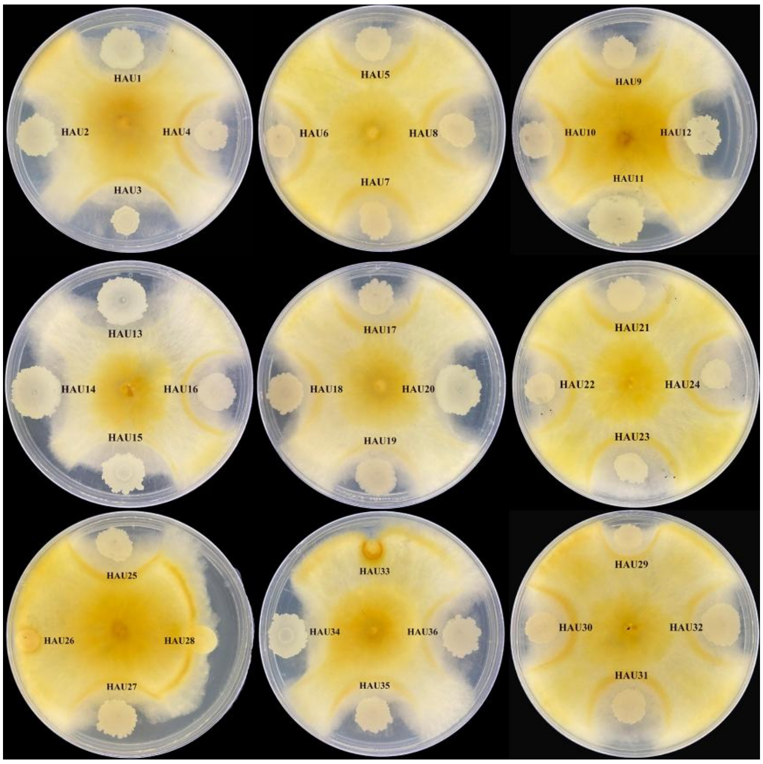

Supplementary Figure 1: Screening of antagonistic strains to *F. graminearum*.

A

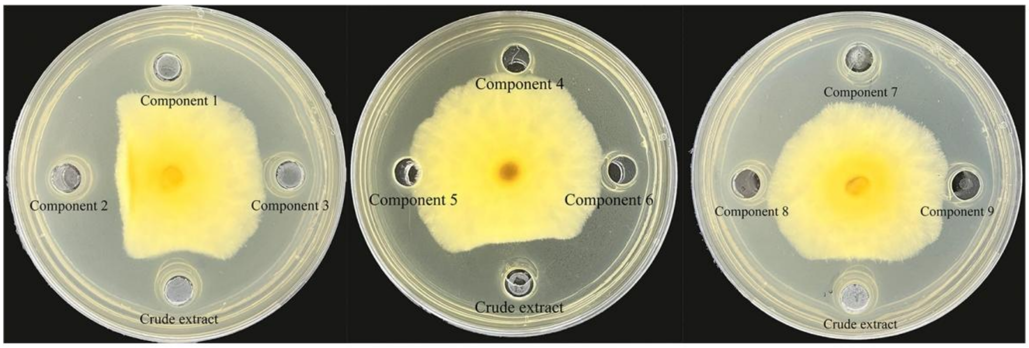

B

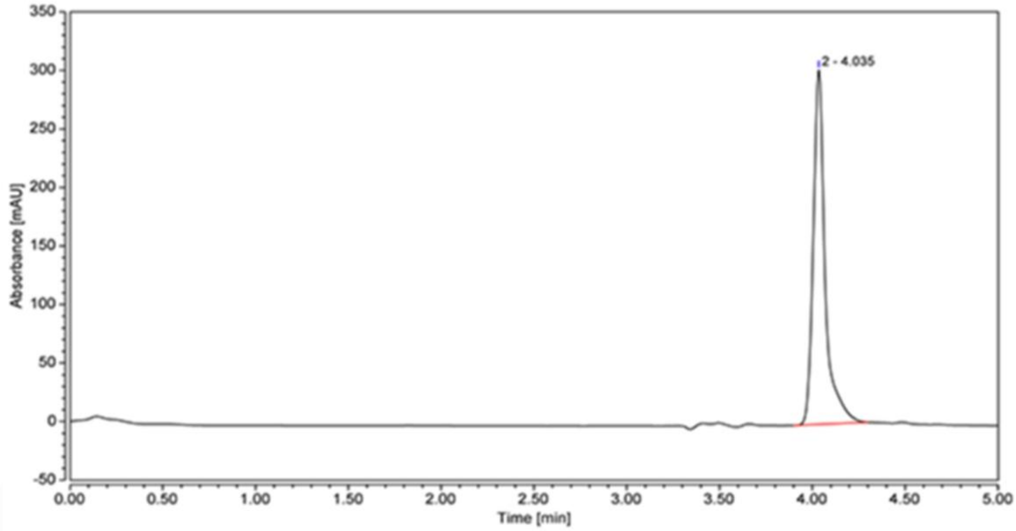

Supplementary Figure 2: Isolation of antibacterial compounds from strain HAU3. A: B acteriostatic effect of each component; Note: Concentration in each well is 100 μg/mL, wi th a volume of 100 μL per well; B: Purity analysis of component 2 by HPLC.

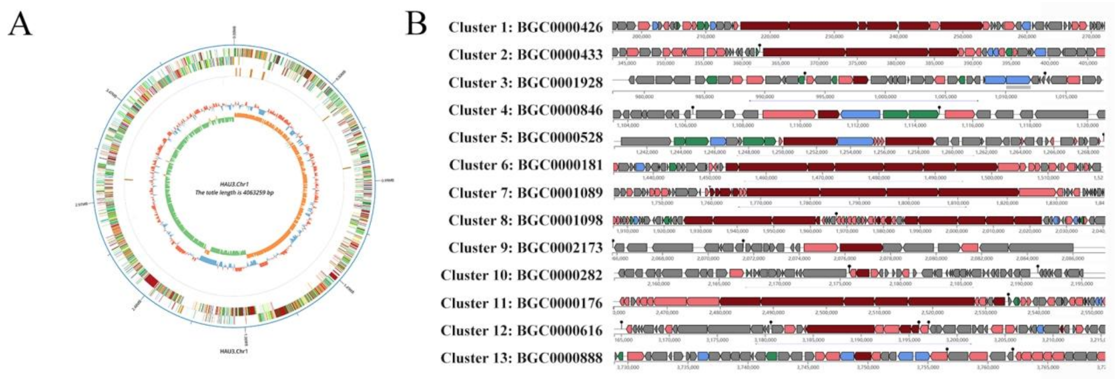

Supplementary Figure 3: Whole genome sequencing analysis of strain HAU3. A: The complete genome map of HAU3; note: the outermost circle represents the genomic sequen ce location coordinates. Moving inward, the layers represent the following: Gene functional annotation results (including COG/KOG annotation information as per the actual project), ncRNA, genomic GC content (calculated using a window size of chromosome length/1000 bp and step size of chromosome length/1000 bp). B: Antismash predicted the stimulating metabolite gene cluster of strain HAU3.
